# Bioactive peptides from venoms against glioma progression

**DOI:** 10.3389/fonc.2022.965882

**Published:** 2022-08-29

**Authors:** Bernarda Majc, Metka Novak, Tamara T. Lah, Igor Križaj

**Affiliations:** ^1^ Department of Genetic Toxicology and Cancer Biology, National Institute of Biology, Ljubljana, Slovenia; ^2^ Jožef Stefan International Postgraduate School, Ljubljana, Slovenia; ^3^ Department of Molecular and Biomedical Sciences, Jožef Stefan Institute, Ljubljana, Slovenia

**Keywords:** cancer, glioma, glioblastoma stem cell, venoms, bioactive peptides, therapy, cancer hallmarks

## Abstract

Venoms are complex mixtures of different molecules and ions. Among them, bioactive peptides have been found to affect cancer hallmarks, such as cell proliferation, cell invasion, cell migration, and can also modulate the immune response of normal and cancer-bearing organisms. In this article, we review the mechanisms of action on these cancer cell features, focusing on bioactive peptides being developed as potential therapeutics for one of the most aggressive and deadly brain tumors, glioblastoma (GB). Novel therapeutic approaches applying bioactive peptides may contribute to multiple targeting of GB and particularly of GB stem cells. Bioactive peptides selectively target cancer cells without harming normal cells. Various molecular targets related to the effects of bioactive peptides on GB have been proposed, including ion channels, integrins, membrane phospholipids and even immunomodulatory treatment of GB. In addition to therapy, some bioactive peptides, such as disintegrins, can also be used for diagnostics or are used as labels for cytotoxic drugs to specifically target cancer cells. Given the limitations described in the last section, successful application in cancer therapy is rather low, as only 3.4% of such peptides have been included in clinical trials and have passed successfully phases I to III. Combined approaches of added bioactive peptides to standard cancer therapies need to be explored using advanced GB *in vitro* models such as organoids. On the other hand, new methods are also being developed to improve translation from research to practice and provide new hope for GB patients and their families.

## 1 Introduction

### 1.1 Bioactive peptides as therapeutic compounds

Bioactive peptides are produced by a wide variety of organisms, from bacteria to plants and higher animals, such as snakes, cone snails, spiders, scorpions and insects. One of their main functions is to defend the producing organism against predators. Biologically and pharmacologically active peptides, here after referred to as “bioactive peptides,” are usually 2–20 amino acid residues (AA) long, although some have been reported to be longer (1). These peptides exhibit a spectrum of pharmacological effects on human organism, of which beneficiary to the homeostasis and/or counteracting the disease have always been attractive objects of research. Structural and functional studies of natural toxins have revealed physiological and pharmacological mechanisms at the cellular and molecular levels. With our growing understanding of their pathophysiological actions, possibilities to employ bioactive peptides as therapeutics emerged (2).

In contrast to small molecule drugs, bioactive peptides generally have narrower target specificity and higher efficacy. Because of their unique selectivity combined with high potency, natural peptide toxins are attracting considerable attention in drug discovery (2). An increasing number of bioactive peptides are moving from laboratory to clinical trials. The balance of therapy-related toxin research is shifting from the development of classical anti-toxins to the development of bioactive peptides as drugs to treat various diseases, including cancer.

Venoms are complex mixtures of different molecules and ions. Among them are bioactive peptides with toxic effects that usually bind to receptor proteins in target organisms to induce adverse reactions (3). Such peptides target signaling pathways in normal and pathological conditions and cause either harmful or beneficial effects in the organism (4). Based on the physiological targets of venom toxins, we distinguish neurotoxins, myotoxins, cardiotoxins and hemotoxins. Venom toxins can also target catalytic functions of various enzymes and act as inhibitors or activators. Based on their site of action, their therapeutic effect can be among others analgesic, neuroprotective, cardioprotective, or anti-cancer (5). A special group of molecules from venoms with anti-cancer activity are venom peptides, on which this review is focused. With their highly specific and selective effects on cancer cells, venom bioactive peptides have been found to affect cancer hallmarks, such as cell proliferation, cell death, cell invasion and cell migration. In addition, they have also been found to modulate the immune response of normal and cancer-bearing organisms (6).

### 1.2 Hallmarks of cancer

**Figure 1:**
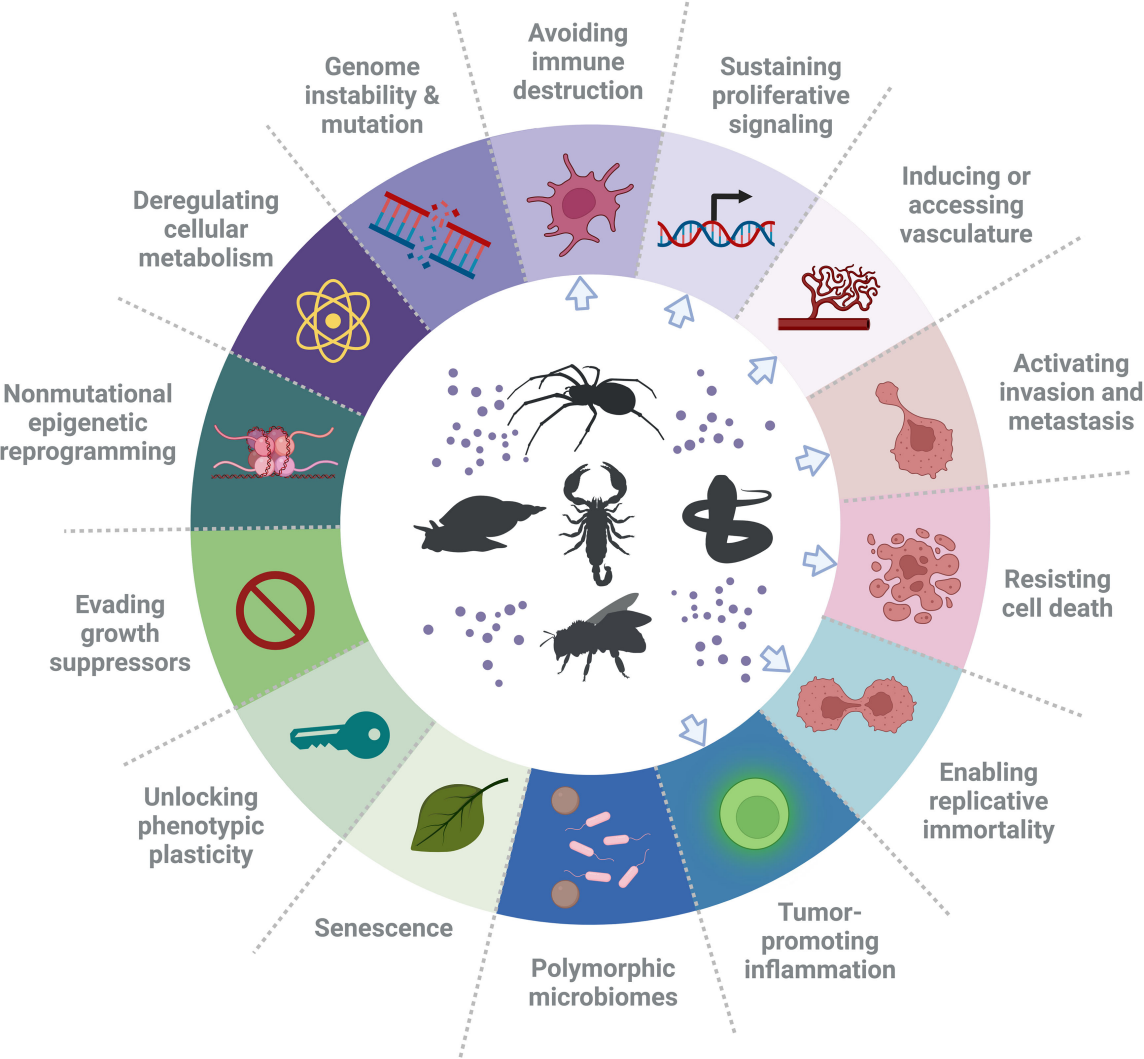
Hallmarks of cancer and suggested types of treatments. In the ring, the hallmarks of cancer, as defined by Hanahan and Weinberg (9), are presented. These hallmarks have been identified as therapeutic targets for new anti-cancer drugs. Noteworthy, there are examples of peptides that have an effect on a single cancer hallmark in various cancers and also have an effect on glioblastoma, or have a specific effect on one cancer. However, a single bioactive peptide can affect more than one hallmark (e.g. invasion and angiogenesis). Blue arrows point at the hallmarks of cancer that have been affected by venom peptides. The figure was generated using BioRender online tool, adapted from “Hallmarks of cancer: circle” [BioRender.com (2022)].

Cancer is one of the major causes of death worldwide. It is anticipated that its burden will rise by 47%, from 19.3 million new cases in 2020 to 28.4 million in 2040 (7). According to Hanahan and Weinberg (8), the term “hallmarks of cancer” refers to ten key features of cancer cells. These are sustaining proliferative signaling, evading growth suppressors, avoiding immune destruction, enabling replicative immortality, inducing tumor-promoting inflammation, activating invasion and metastasis, promoting vascular development, enhancing genome instability and mutation, resisting cell death and deregulating cellular metabolism. In a 2022 review (9), Hanahan additionally defined four newly emerged traits of cancer cells, termed “enabling characteristics”, including unlocking phenotypic plasticity, non-mutational epigenetic reprogramming, polymorphic microbiomes and senescence. Therefore, a total of fourteen cellular attributes currently outline key processes of cancer progression (9) and thus also define key cellular/molecular targets to inhibit them and normalize physiology. This helps predicting properties of novel substances to treat human cancer, which include venom-derived bioactive peptides (**Figure 1**).

Several venom peptides have been evaluated for their effects on various hallmarks of cancer. For example in different types of cancer, including glioblastoma (GB), the AGAP peptide from scorpion venom has been shown to decrease cell proliferation and inhibit angiogenesis, tissue invasion, and metastasis (10), whereas chlorotoxin inhibits invasion and metastasis (11), cell death resistance, and angiogenesis (12). Iberiotoxin, another scorpion toxin, has been shown to balance deregulated cell proliferation (13), while melittin, a peptide from the honeybee venom, stimulates DNA damage, activating the intrinsic pathway of apoptosis and thus causing the death of most resisting cancer cells (14–18). Another example are disintegrins from snake venom, which reduce angiogenesis, tumor inflammation and inhibit invasion and metastasis (19). These and other venom peptides targeting hallmarks of other cancers are a topic of several recent review papers (5, 19, 20). Elsewhere, anti-tumor effects of venom peptides in various types of cancer at the molecular and cellular level are described (3, 10, 21). In this systematic review, we are focusing on peptides derived from venoms that affect GB progression and are being developed as potential anti-GB therapeutics.

### 1.3 Glioblastoma and resistance to therapy

As presented by Louis et al. (22), there is a variety of central nervous system (CNS) cancers. Gliomas are the most frequent primary brain tumors in adults. The new classification from the World Health Organization (WHO) distinguishes four grades of astrocytoma. *De novo* GB is defined as a diffuse astrocytic glioma WHO grade IV without mutations in the isocitrate dehydrogenase (IDH) genes (23). GB is the most common human glial tumor, occurring in 5 to 7 adults per 100,000 per year (24). Necrosis, vascular proliferation and pleomorphism are the main histological features of GB. The most common factor associated with poor prognosis is diffuse infiltration of highly invasive single GB cells into the brain parenchyma, which makes complete resection of the tumor impossible, thus causing resistance to therapy.

GB stem cells (GSCs) are the second factor responsible for the high therapy resistance of GB. These cancer stem cells (CSCs) generally express high levels of DNA damage repair proteins as well as the ABC family transporters that render GSCs less vulnerable to radiation and chemotherapy. For this reason, GSCs are far more resistant to cytotoxic drugs compared to differentiated GB cells. In addition to their relatively high genetic stability, the specific location of CSCs in the so-called tumor “niches” and the hypoxic environment, which causes a low proliferation rate of CSCs, are also of great importance for their therapy resistance (25). GSC niches in the brain are often located in the subventricular zone (26). The brain parenchyma is a relatively common secondary metastatic site because of its permissive microenvironment which consists of brain-specific stromal cells and the extracellular matrix (ECM) into which CSCs from other organs can migrate (27). The metastatic process is triggered by paracrine interactions with CSC receptors, presumably after acquiring the invasive phenotype *via* an epithelial-to-mesenchymal (EMT)-like transition. Similarly, we assume that GSCs undergo proneural-mesenchymal transition (PMT) (28) to metastasize to other organs, not such a rare process, as previously assumed (27). Taken together, there is a general agreement that prolonged GB patient survival can only be achieved by combined treatments targeting GSCs with synthetic drugs, antiangiogenic agents and other targeted biological therapeutics, gene therapy, promising immunomodulation (29) and lastly, possibly bioactive peptides.

#### 1.3.1 Current glioblastoma therapeutic strategies

The current standard therapy for GB includes surgery and a combination of radiation and the alkylating agent temozolomide treatment using various protocols (30). However, the beneficial effect of temozolomide is usually, but not always, limited to patients with *MGMT* promoter-methylated GB (31). The recent consensus review of the Society for Neuro-Oncology (SNO) and the European Association of Neuro-Oncology (EANO) (32) discusses in detail the current medical treatment options and supportive care of GB patients. In addition to surgery and postoperative treatment, radiotherapy and patient-specific treatment with small molecule chemotherapeutic agents, monoclonal antibodies targeting either cancer cells or stromal cells have been described in detail. Regarding the latter, bevacizumab that target vascular endothelial growth factor (VEGF) attracting endothelial cells in (*MGMT* promoter-unmethylated) recurrent GB, is frequently, but unsuccessfully used to prevent angiogenesis. Increased invasion of GB cells after such treatment has been observed and is mainly due to induction of hypoxic areas within the tumor, being the main obstacle of bevacizumab efficacy (33).

Immunotherapies may also be considered to overcome the immunosuppressive GB tumor microenvironment (TME), which is defined as immunologically ‘cold’. This means that GB TME lacks effector lymphocytes and is infiltrated with large amounts of suppressive myeloid cells and regulatory T cells (Tregs). Immunosuppression can also be caused by corticosteroids that are applied to reduce tumor-associated edema, as standard treatment of GB patients (34). Despite intensive research, current immunotherapy strategies, including chimeric antigen receptor CAR T cells and natural killer (NK) cells, oncolytic viruses (OVs) and vaccines, have not achieved clinically-relevant effects (35). Therapies targeting specific cytokines (such as TGF-β), receptors (colony-stimulating factor receptor) or immune checkpoint proteins (PD-1, PDL-1), all related to GB-immune cells cross-talks, have not shown much success yet in clinical trials (35).

New therapeutic molecules aimed to overcome GB resistance are constantly being explored and added to standard treatment. Anti-cancer bioactive peptides are promising to be more efficient in targeting GB and to be less harmful as conventional GB therapies. Peptides used for targeting and therapy of GB are classified into different categories, such as tumor-homing peptides, peptides targeting aberrant cellular signaling pathways and cell-penetrating peptides (36). The use of peptides from venoms as adjuvants may open new perspectives for treatment of GB (2, 4, 36).

## 2 Molecular background of action of venom peptides on glioblastoma

**Figure 2:**
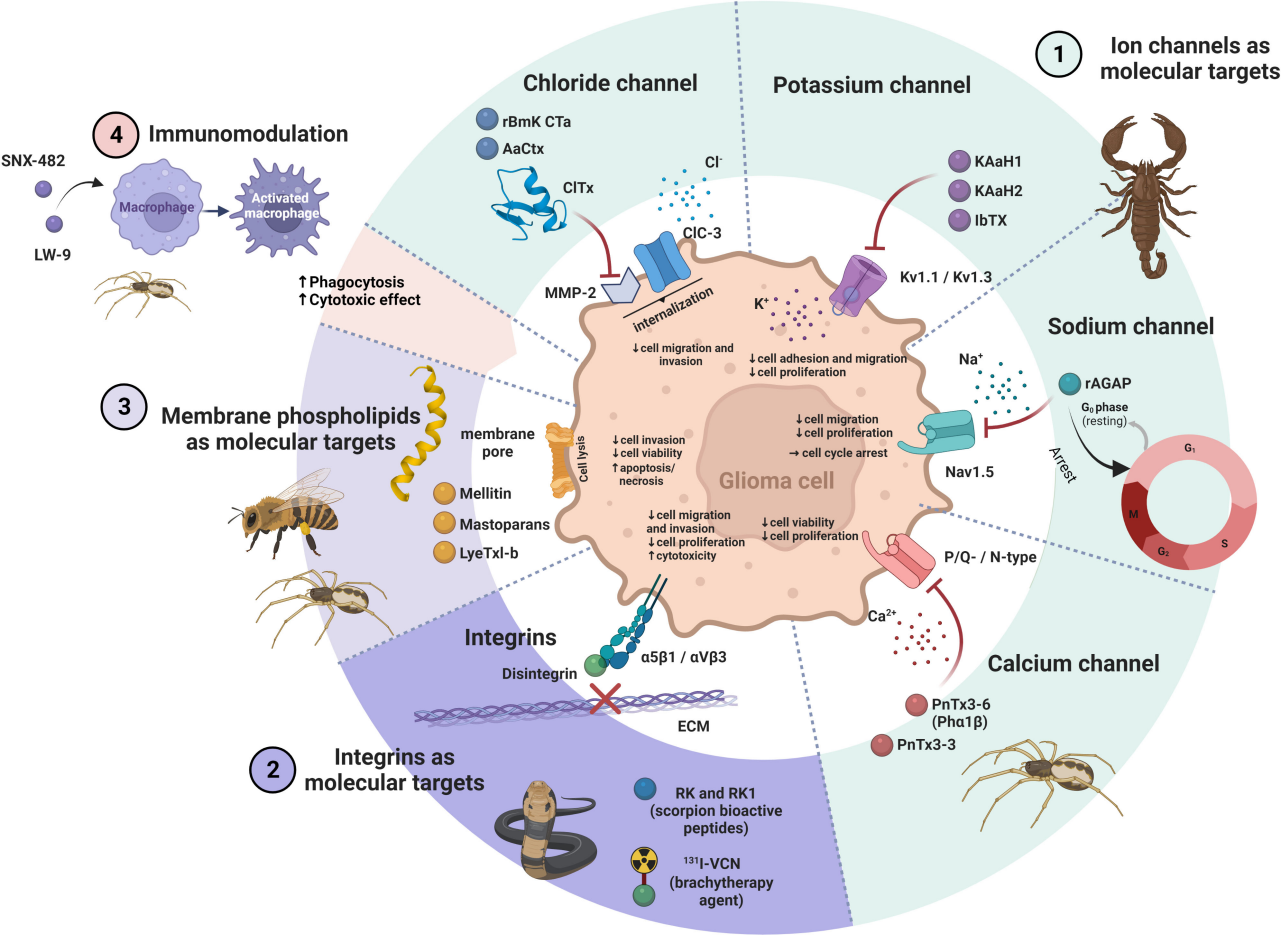
The ways of action of venom peptides on glioma cell. 1.) Venom peptides block different ion channels in glioma cells, such as Cl^−^, K^+^, Na^+^ and Ca^2+^ resulting in a decrease in viability, proliferation and invasion of the cells. 2.) Venom peptides (*e.g.* disintegrins) bind to integrins and inhibit their interaction with the ECM. This impairs migration and proliferation of glioma cells and induce cell death. 3.) Venom peptides that interact with phospholipids in the plasma membrane of glioma cells disturb its integrity with the result of perforation of the membrane leading to cell lysis and death. 4.) The immunomodulatory effects of venom peptides on macrophages may enhance their cytotoxic and phagocytic effect on glioma cells. The figure was created using BioRender online tool (BioRender.com).

Bioactive peptides from venoms are known to play their therapeutic role through different mechanisms of action, which makes them unique agents compared to existing commercial drugs (10). They act selectively on cancer cells without being harmful to normal cells (37). Various molecular targets have been proposed to be related to the effect of venom peptides on GB, including ion channels, integrins and membrane phospholipids. The effects of venom peptides on GB have also been related to immunomodulation (**Table 1** and **Figure 2**).

**Table 1:** Bioactive peptides from venoms to resist GB.

Bioactive peptide	Molecular target/Mechanism	Effect on GB	Venom	Study	References
**Chlorotoxin (ClTx)**	Cl^-^ channels	↓ migration, invasion	Death stalker scorpion (*Leirus quiquestriatus*)	*in vitro*, *in vivo*, Translated into the clinics	(11, 38–41)
**rBmK-CTa**			Chinese scorpion (*Mesobuthus martensii*)	*in vitro*	(42)
**AaCtx**			Southern man-killer scorpion (*Androctonus australis*)	*in vitro*	(43)
**KAaH1**	K^+^ channels	↓ migration, adhesion	Southern man-killer scorpion (*Androctonus australis*)	*in vitro*	(44)
**KAaH2**		↓ proliferation		*in vitro*	
**Iberiotoxin (IbTX)**		↓ proliferation	Eastern Indian red scorpion (*Hottentotta tamulus*)	*in vitro*	(13, 45, 46)
**AGAP**	Na^+^ channels	↓ migration ↓ proliferation cell cycle arrest	Chinese red scorpion *(Buthus martensii*)	*in vitro*	(47, 48)
**PnTx3-6 or Phα1β**	Ca^2+^ channels	↓ proliferation ↓ viability	Wandering spider (*Phoneutria nigriventer*)	*in vitro*, *In vivo*	(49)
**PhTx3-3**				*In vivo*	
**Disintegrins**	Integrins	↓ invasion	Saharan horned viper (*Cerastes cerastes*)	*in vitro*	(50)
		↑ cytotoxicity ↓ migration ↓ proliferation	Anatolian meadow viper (*Vipera anatolica*)	*in vitro*	(51)
**RK and RK1**		↓ migration ↓ proliferation	Common yellow scorpion *(Buthus occitanus tunetanus)*	*in vitro*	(52)
**Melittin**	Membrane phospholipids	↓ invasion ↓ proliferation ↑ apoptosis	Honeybee (*Apis mellifera*)	*in vitro*	(18, 53)
**Mastoparan 1 (MP1)**		↓ viability ↑ necrosis	Wasp(*Polybia paulista*)	*in vitro*	(54)
**Mastoparan X (MPX)**			Yellow hornet (*Vespa xanthoptera)*		
**Mast cell degranulating peptide (HR1)**			Oriental hornet (*Vespa orientalis)*		
**LyeTxl-b**		↑ necrosis and necroptosis	Wolf spider *(Lycosa erythrognata)*	*in vitro*	(55)
**Fractions F1–F3, and subfractions SF1–SF11**	Immunomodulation	↑ number of monocytes in blood of xenogeneic mice and number of macrophages infiltrating tumor	Wandering spider (*Phoneutria nigriventer*)	*in vitro*, *In vivo*	(56–58)
**LW-9**		↑ cytotoxic and phagocytic activity of macrophages		*in vitro*	(59)
**SNX-482**		↑ activation polarized (non-activated) M0 macrophages	African tarantula (*Hysterocrates gigas)*	*in vitro*	(60)

↓ decreased, ↑ increased.

### 2.1 Ion channels as targets of venom peptides

Ion channels are specialized transmembrane proteins that regulate the flow of ions into cells and/or out of cells and their intracellular compartments, respectively. As a consequence of ion flow, specific intracellular signaling may be triggered. Ion channel dysfunction is a key feature of numerous pathologies. In the human genome, 330 genes encode ion channels, which differ in their selectivity for ions and their gating mechanism (61, 62). Ion channels are classified on the basis of the ion they conduct to chloride (Cl^−^), sodium (Na^+^), potassium (K^+^) and calcium (Ca^2+^) channels, or according to their gating characteristics, this is, how their opening and closing is controlled. Ion channels are important for cell migration, proliferation, apoptosis and regulation of gene expression in cancer cells (63). Genome profiling has shown that genes encoding ion channels are among the most commonly mutated genes in GB (64, 65). Abnormal ion channel function has been associated with migration (66, 67) and proliferation of GB cells (13). For example, Pollak et al. (68) identified higher expression of several ion channels in GSCs compared to neural stem cells (NSCs) that were associated with reduced survival of patients. Genetic silencing or blocking of these channels reduced the viability of GSCs, indicating the therapeutic potential of ion channel blockers in GB.

#### 2.1.1 Cl^−^ channels

Cl^−^ channels play important roles in physiological processes, such as cell membrane potential maintenance, cell volume regulation, cell proliferation and intracellular pH control. They are either voltage-sensitive ClC channels, Ca^2+^-activated channels, high conductance channels, cystic fibrosis transmembrane conductance regulators (CFRTs), or volume-regulated channels (69).

There is growing evidence that Cl^−^ channels contribute to tumor development and progression, with the ClC subfamily playing an important role in GB cell migration and invasion (70). ClC-2, ClC-3 and ClC-5 are overexpressed in GB. Higher expression of these three Cl^−^ channels results in increased transport of Cl^−^ ions into the GB cell leading to changes in cell shape and size to facilitate cell invasion (71). In a recent study, an association was demonstrated between high ClC-3 expression and increased GB cell invasion promoted by NF-κB signaling, that was associated with shorter patient survival (72).

Chlorotoxin (ClTx), a 36 AA basic peptide isolated from the venom of the death stalker scorpion *Leirus quiquestriatus* (73), was the first identified Cl^−^ channel blocker that impaired GB cell growth and invasion (38). NMR analysis of ClTx revealed that its α-helix is connected by three disulfide bonds to antiparallel β-sheets (39). Due to its low molecular mass, it can cross the blood brain barrier (BBB) (40) and then specifically binds to malignant cancer cells. In addition to the voltage-gated Cl^−^ channels, ClTx interacts also with annexin-2 and the secreted matrix metalloproteinase-2 (MMP-2). In GB, MMP-2 is involved in degradation of the ECM, whereas the ClC-3 ion channel is associated with regulation of the cell shape and volume, both facilitating its invasive behavior (66). It has been suggested that ClTx binds to the MMP-2 in glioma cells (11). This binding leads to the endocytosis of the complex of ClTx, MMP-2 and ClC-3 channel, located close to MMP-2, resulting in reduced cell migration (10). ClTx has been shown to have some anti-invasive and anti-angiogenic effects *per se* (41), although it is not cytotoxic to neither cancer cells nor healthy cells. Due to its high specificity for cancer cells, ClTx has been demonstrated to be very promising as tumor-labelling and tumor-guiding molecule. Thus, several drug vehicles for chemotherapy and gene therapy have been coated with ClTx to achieve specific targeting (40). In a very recent study, the authors designed a ClTx-CAR T cell to achieve specific tumor targeting. In this way, by more efficient GB targeting, tumor regression in orthotopic GB xenograft models was obtained (74). Radioactively labelled ClTx (^125^I- or ^131^I-ClTx) is a promising substance for radiotherapy of postoperative brain tumors (75, 76). Fluorescently labelled-ClTx, termed BLZ-100 (a.k.a. tozuleristide or Tumor Paint^®^), facilitated demarcation of cancer foci from adjacent normal tissue (77–79). The recombinant ClTx, designated TM-601, reached phase III clinical trials (NCT00040573), BLZ-100 phase I trials were successfully concluded (NCT02234297) and ClTx-CAR T cells are currently in phase I clinical trials (NCT04214392). There are other scorpion venom peptides that bind Cl^−^ channels, which are studied for their anti-GB applications. For example, the ClTx-like peptide rBmK-CTa from the venom of Chinese scorpion (*Mesobuthus martensii*), which has been shown to inhibit the growth and proliferation of human glioma cells (42) and the AaCtx peptide from the venom of southern man-killer scorpion (*Androctonus australis*), which prevented invasion and migration of human glioma cells (43).

#### 2.1.2 K^+^ channels

There are four classes of K^+^ channels, namely voltage-gated K^+^ channels (K_v_), which are the largest group of K^+^ channels, Ca^2+^-activated K^+^ channels (K_Ca_), which are further divided into big conductance (BK), intermediate conductance (IK) and small conductance (SK) channels, inward rectifying K^+^ channels (K_ir_) and two-pore-domain K^+^ channels (K2P) (80).

In cancer, altered expression of K^+^ channels has been found and their involvement in the regulation of cancer cell proliferation and apoptosis have been shown as well (81). For example, K_Ca_ channels, such as K_Ca_3.1, and the BK channels are overexpressed in 32% of glioma patients and this correlates with poor survival, due to increased glioma cell invasion (82). Other types of K^+^ channels include K_ir_ channels, such as K_ir_4.1 and K_ir_4.2, that are downregulated in glioma cells and K_v_ channels, such as hERG (human Ether à go-go Related Gene channel) that has been shown to be overexpressed in GB (83). Other K_v_ channels, *e.g.* K_v_1.5, appear to be associated with prolonged survival of cancer patients (84).

Different peptides from scorpion, snake, bee and sea anemone venoms show high affinity toward specific classes of K^+^ channels (85). Among these peptides, CSα/β toxins from scorpion venom have been shown to block K^+^ channels and to inhibit various hallmarks of cancer. KAaH1 and KAaH2 are two peptides from the scorpion venom of *Androctonus australis* (44) that block specific K_v_ channels in GB. KAaH1 has been shown to block K_v_1.1 and K_v_1.3 channels, resulting in inhibition of migration and adhesion of GB U87 cells, whereas KAaH2, which only slightly affects the K_v_1.1 channel, alters EGFR signaling and decreases U87 cell proliferation (86). These results support targeting of K^+^ channels as anti-cancer therapeutic approach. KAaH1 and KAaH2 may also be used as specific tools to study the mechanisms of K_v_ channels in various types of cancer and especially in GB (86). Another scorpion venom toxin is iberiotoxin (IbTX), purified from the Eastern Indian red scorpion (*Hottentotta tamulus*), which selectively inhibits BK channels. Studies have shown that IbTX arrests glioma cells in the S phase of the cell cycle and impairs cell proliferation (13, 45, 46), whereas other studies found the opposite these results and showed that BK channels are not required for cell proliferation (87).

#### 2.1.3 Na^+^ channels

Two groups of Na^+^ channels are defined, the voltage-gated Na^+^ channels (VGSC or Na_v_) and the epithelial Na^+^ channels (ESC). Na_v_ channels are expressed in the nervous system and other excitable or non-excitable cells. They are responsible for the generation of action potentials (88). Nine Na_v_ isoforms are known (Na_v_1.1 to Na_v_1.9), differently spread in the human body. Na_v_1.1, 1.2, 1.3 and 1.6 are found in the CNS, whereas Na_v_1.7, 1.8 and 1.9 are mainly found in the peripheral nervous system. Na_v_1.4 and Na_v_1.5 are expressed in adult skeletal muscle and cardiac muscle, respectively (89). Relatively high expression levels of Na_v_ channels have been found in some types of non-excitable cell, including immune cells, fibroblasts and cancer cells (90). Multiple isoforms of Na_v_ channels are expressed in different types of cancer cells (90), whereas their expression has not been detected in corresponding normal cells. The higher the expression of Na_v_ channels, the higher was the motility of cancer cells, their invasiveness and metastatic potential. However, the mechanism behind these traits and the impact of increased expression of Na_v_ channels in the cells is still unknown. Gene expression analyses have shown that Na_v_ channels are upregulated in GB tissues. In addition, mutations in Na_v_ channels have been found in at least 90% of GB samples correlating with shorter patient survival (91).

Recent preclinical data suggest that pharmacological targeting of Na_v_ reduce invasion and metastasis in breast cancer mouse models (92), although the utility of these inhibitors in anti-metastatic therapy has yet to be proven in clinical studies. One group of venom peptides targeting Na^+^ channels are Na^+^ channel scorpion toxins (NaScTxs) (93). These peptides are classified into α- and β-type toxins according to their modes of binding and action (10, 94). So far, only few NaScTxs have been evaluated for their effects on cancer cells as is summarized by Srairi-Abid (10). Among them, a peptide AGAP, isolated from the venom of the *Buthus martensii* scorpion, was tested on glioma cells and showed antitumor and analgesic effects (47). AGAP is a β-type long-chain toxin, consisting of 66 AA, cross-linked by four disulfide bridges (95). Its recombinant form (rAGAP) inhibited proliferation of SHG-44 human malignant glioma cells by arrest in the G1 phase of the cell cycle and impaired migration of SHG-44 cells. Recombinant AGAP has been shown to affect protein kinase B (AKT), p38 mitogen-activated protein kinase (p-38), extracellular signal-regulated kinases (Erk1/2), c-Jun kinases signaling pathways and reduced VEGF and MMP-9 expression levels (48). The authors suggested that AGAP induces these effects by inhibiting the Na_v_1.5 channel, causing a disruption of intracellular ion homeostasis (48).

#### 2.1.4 Ca^2+^ channels

Voltage-gated Ca^2+^ channels (VGCC) are transmembrane proteins that are activated upon membrane depolarization, allowing Ca^2+^ to enter the cell and trigger many physiological events. VGCCs are classified into 6 types, referred to as L-, P/Q-, N-, R- and T-type, depending on channel conductance, kinetics, cell distribution and sensitivity to specific blockers (96).

There is increasing evidence that Ca^2+^ channels play an important role in cancer cell proliferation, resistance to apoptosis, drug resistance and invasion (97). GB-related Ca^2+^ channels include P/Q-type (Ca_v_2.1), N-type (Ca_v_2.2) and T-type (Ca_v_3.1–3.3) channels, which are abundant in the central nervous system (98). It has been shown that the T-type Ca_v_3.2 channel is highly expressed in GSCs and that its expression correlated with patient survival. Blocking these channels by the FDA-approved drug mibefradil (T-type channel blocker) or RNAi-mediated attenuation of the channel enhanced the effect of temozolomide and consequently inhibited cell growth and induced cell death of GSCs (99). Moreover, the use of tetralol derivates (T-type Ca^2+^ channel blockers) remarkably delayed tumor progression in GB mouse xenografts (100). Targeting the dysregulated Ca^2+^ channels may thus represent a promising chemotherapy for GB treatment.

Venoms are rich sources of potent and selective VGCC peptide inhibitors. So far, only two peptides targeting VGCCs have been tested on GB. PnTx3-6 (also Phα1β), a peptide from the venom of the wandering spider (*Phoneutria nigriventer*), is a VGCC blocker with a characteristic ICK structural motif (101). Without being toxic, it exhibited potent analgesic effect in rat models of inflammatory and neuropathic pain as well as an anti-nociceptive effect (20). The effect of PnTx3-6 on GB progression was evaluated in the study by Nicoletti et al. (49). PnTx3-6 and the structurally similar VGCC blocker, PhTx3-3 induced a significant reduction in cell proliferation and viability of GB cell lines (M059J, U138MG and U251MG). Moreover, PnTx3-6 and its recombinant form, CTK 01512-2, reduced tumor growth in mouse GB model and increased the number of activated astrocytes and microglial cells in the peritumoral area.

### 2.2 Integrins as targets for venom peptides

Integrins belong to the family of transmembrane receptors involved in cellular adhesion with specific elements of the ECM, such as fibronectin, vitronectin, laminin and collagen. Upon binding an extracellular ligand, integrin dimers activate downstream signaling pathways that regulate many cellular processes, including cell survival, migration, proliferation and inflammatory activity (102). Integrins are heterodimeric complexes of non-covalently associated α- and β-subunit. There are at least 24 integrin receptors. They are composed of different combinations of the 18 α- and 8 β-subunits (103) and have different ligand-binding properties and tissue distribution patterns. The specificity of integrins to interact with ECM proteins depends on the presence of their conserved tripeptide motif, usually Arg-Gly-Asp (RGD), but also Met-Leu-Asp (MLD) or Arg/Lys-Thr-Ser (R/KTS), to which they bind. A number of integrins are frequently upregulated in various types of cancers (104), including GB (105). According to The Cancer Genome Atlas (TCGA) datasets, integrin subtypes, such as α_v_β_3_ and α_v_β_5_, are upregulated in GB and associated with poor patient prognosis. Integrin expression patterns have also been shown to correlate with GB subtypes. The mesenchymal subtype, for example, exhibits global integrin overexpression, except for β_8_ and α_6_, which are mainly overexpressed in the classical GB subtype (105). Integrin α_6_ was also found to be highly expressed in GSCs, making this protein a candidate marker for GSC targeting. Since α_6_ integrin is involved in the self-renewal and proliferation of GSCs, it is also a potential therapeutic target for GB (106). Several integrin-binding peptides from venoms have shown anti-cancer activity by binding to integrins, inhibiting their interaction with the ECM and hence their name – disintegrins (107).

Disintegrins are small (40–100 AA), cysteine-rich and non-enzymatic peptides that bind to and inactivate integrins on the surface of cancer cells and normal cells (108). Disintegrins are mainly found in snake venoms (109) and are usually generated by proteolytic processing of the snake venom metalloproteases (SVMP) (108). Of RGD-, MLD- and R/KTS-containing disintegrins, the first has been most frequently studied (110). RGD-disintegrins exhibit anti-cancer effects by inhibiting cell adhesion to the ECM (111) and as a consequence cell migration and invasion (110). For example, disintegrin from the venom of the Saharan horned viper (*Cerastes cerastes*) suppressed invasion of GB U87 cells (50). Cytotoxic activity of disintegrins from the Anatolian meadow viper (*Vipera anatolica*) venom has been demonstrated in U87 cells (51). The proposed mechanism behind the disintegrin-induced cytotoxicity was their binding to α_5_β_1_ and α_v_β_3_ integrins, which significantly reduced survival, proliferation and migration of GB U87 cells (IC_50_ value 0.51 ± 0.04 mg/ml). Radiolabeled disintegrins (^131^I-VAT) were used to analyze their uptake by GB U87 cells (112). Based on these results, it was concluded that ^131^I-VAT could be employed as an agent to facilitate imaging of GB. ^131^I-labeled disintegrin vicrostatin (^131^I-VCN) has also been shown to be suitable for imaging GB. VCN is a recombinant disintegrin derived from contortrostatin, a disintegrin isolated from the venom of the snake *Agkistrodon contortrix* and viperid disintegrin echistatin (*Echis carinatus*) (21). Two glioma mouse models, the orthotopic xenograft glioma model and the syngeneic GL261 mouse model, were used to test ^131^I-VCN as a brachytherapy agent. It was found that the therapy prolonged survival of treated animals (113). Moreover, the combination of temozolomide and ^131^I-VCN showed better therapeutic results than the combination of radiotherapy and temozolomide. Therefore, VCN has been shown as a suitable delivery system to target GB for radiotherapy. RGD-disintegrin, cilengitide, the first anti-angiogenic small molecule that selectively blocks integrins α_v_β_3_ and α_v_β_5_ (114), completed a phase III clinical trial for treatment of recurrent glioblastoma (NCT00093964).

From the *Buthus occitanus tunetanus* scorpion venom, short peptides targeting integrins, have been recently purified. The peptides, RK and RK1, 17 and 14 AA in size, suppressed migration and proliferation of GB U87 cells, possibly by interacting with integrins α_1_β_1_ and/or α_v_β_3_ (52). RK1 contains two distinct AA motifs: KSS that interacts with α_1_β_1_ and ECD with affinity for α_v_β_3_ integrin. The KSS motif does not appear to be involved in the disintergrin function of RK, whereas the ECD motif inhibits α_v_β_3_ to prevent cancer cells from spreading (3). Both peptides represent the first members of a new group of scorpion venom peptides with anti-tumor potential and may open a new perspective in drug development for treatment of GB.

### 2.3 Cell membranes as targets of venom peptides

Integrity of the plasma membrane is critical for functioning of all living cells (115). Several peptides from venoms can induce membrane disorganization and loss of its function. Cancer cells contain a higher percentage of negatively charged lipids in their membranes compared to normal cells so they interact with the positive residues of a subset of natural peptides (cationic amphipathic peptides) more readily, which therefore represent an effective source of potential anti-cancer agents (116).

One of those is melittin from the venom of honeybee (*Apis mellifera*) (117). Melittin is a phospholipid-interacting and lytic peptide of 26 AA. It forms a long helical structure with a hydrophilic N-terminus and a hydrophobic C-terminus. Melittin triggers cell lysis by forming membrane pores with an approximate diameter of 4.4 nm, through which then ions and other small molecules leak, ultimately leading to cell lysis (117). The anti-tumor activity of melittin has been demonstrated in various types of cancers (14, 15, 118, 119), including GB (18, 53). The anti-neoplastic activity of melittin includes inhibition of invasion, proliferation and induction of apoptosis in cancer cells. It was shown that it inhibits the PI3K/Akt/mTOR axis in breast cancer (16), MAPK in melanoma (120), JAK2/STAT3 in ovarian cancer (121) and the NFκB signaling pathway in lung carcinoma cells (122). The effect of bee venom (BV) on cell viability, apoptosis, as well as protease MMP-2 expression and activity has been found in GB A172 cells (53). After BV treatment, the authors observed decreased GB cell viability. Moreover, decreased expression and activity of MMP-2 was observed, which was associated with a reduction of invasiveness of GB cells. The exact mechanism by which BV affects MMP-2 is still unknown. The key mechanism of the anti-cancer effect of BV has been attributed to melittin, a major component of the venom (123). In a recent study, the effect of both BV and melittin was evaluated on various GB cell lines (18). It was shown that the peptide induced cell death in GB Hs683, T98G and U373 cells, but also exhibited cytotoxic effects on immune cells of patients (18). The authors demonstrated an increased ratio of the pro-apoptotic proteins Bak and Bax in GB cells that led to apoptosis. Despite the rather non-specific lytic activity and rapid degradation of melittin in the blood, it is still considered as an attractive candidate for anti-cancer therapy (124). Its therapeutic potential is further exploited using gene therapy alone (124), or combined with a suitable delivery system, such as nanoparticles (125).

Mastoparan peptides are the most common constituent of wasp venoms, which adopts a linear, amphipathic α-helical form and disrupt the membranes, eventually leading to cell death (101). Mastoparans, MP1 from the wasp *Polybia paulista*, MPX from the yellow hornet (*Vespa xanthoptera*) and mast cell degranulating peptide HR1 from the Oriental hornet (*Vespa orientalis*), showed anti-cancer activity in GB T98G cells (54). All three peptides consist of 14 AA and adopt an amphipathic structure when bound to the membrane. MP1 has been shown to have broad-spectrum bactericidal and potent anti-cancer activity (126), MPX bactericidal and hemolytic activity (127), and HR1 membrane-permeabilizing activity against bacteria and erythrocytes (128). All three peptides decreased viability of GB cells and induced necrotic cell death, characterized by morphological changes, membrane disruption and an increase in intracellular Ca^2+^ levels. The authors confirmed that the mastoparan activity was the consequence of cell membrane lysis. A direct relationship between membranolytic efficacy of mastoparans and the presence of negatively charged phospholipids, such as phosphatidylserine, in cell membrane has been suggested (54) and opened new prospects for mastoparans as anti-cancer compounds.

Another synthetic peptide LyeTxI-b, derived from the Wolf spider (*Lycosa erythrognata*) venom with membranolytic activity showed anti-cancer activity on GB U87 cells (55). LyeTxI-b was originally synthesized as a potential antibiotic against resistant bacteria. The peptide has a slightly curved amphipathic helical structure consisting of 26 AA (129). The authors indicated that LyeTxI-b induced pore formation and membrane permeabilization of GB U87 cells, followed by necrosis and cell necroptosis (regulated necrosis) (55). Similar to melittin, LyeTxI-b exhibited unfavorable cytotoxicity, including hemolysis and immunotoxicity. Despite these limitations, the peptide represents an interesting prototype to be used as a model for development of new chemotherapeutics.

### 2.4 Venom peptides are immunomodulators

As mentioned above, components of the wandering spider (*Phoneutria nigriventer*) venom (PnV), such as PnTx3-6 and PnTx3-3 peptides, have recently been shown to have anti-GB effects (49). Structurally and functionally uncharacterized peptides isolated from the PnV venom, namely fractions F1, F2, F3 and subfractions SF1–SF11, affected GB cells *in vitro* (56–58) and impaired GB progression in a xenogeneic mouse model *in vivo* (130). However, in this study, PnV venom also affected the TME and especially the local tumor immunosuppressive microenvironment to reactivate anti-tumor immunity, in particular tumor-associated macrophages (TAMs). In GB, TAMs account for up to 40% of the total tumor mass (131), being mostly polarized to the M2 phenotype, which is anti-inflammatory and tumor-supportive, thus promoting carcinogenesis and cancer progression. TAMs stimulate tumor-associated angiogenesis, resistance to chemotherapeutic agents and suppress anti-tumor immunity responses (132). Several pharmacological strategies have been proposed to target TAMs (133), including the use of venom peptides. The above-mentioned PnV fractions were found to increase the number of monocytes in the blood of xenogeneic mice and the number of macrophages infiltrating in the tumor (130). It was demonstrated that the PnV spider venom fraction LW-9 increased cytotoxic and phagocytic activity of macrophages by immunomodulation (59). It was suggested that macrophages were reeducated into a non-TAMs phenotype, resulting in enhanced destruction of cancer cells.

SNX-482 is another peptide with immunomodulatory activity. It was isolated from the venom of the African tarantula (*Hysterocrates gigas*). The peptide consists of 41 AA and was previously described as a selective antagonist of R-type Ca^2+^ channels containing the α1E (Ca_v_2.3) subunit (134, 135). The results of the recent study suggest that SNX-482 activates polarized (non-activated) M0 macrophages by increasing co-stimulatory proteins (CD40, CD68, CD80, CD83, CD86), involved in antigen presentation, whereas an effect of the peptide on polarized M1 and M2 macrophages was not observed. SNX-482 was also shown to upregulate the expression of *CCR4* (C-C motif chemokine receptor 4), *IFNG* (IFN-gamma), *GZMB* (granzyme B) and *PDCD1* (programmed cell death protein 1) genes, which are important for anti-cancer activity. On the other hand, SNX-482 peptide induced macrophage death and decreased the percentage of dead cancer cells. The results of this study suggest that the peptide could be used to activate macrophages for adoptive cell therapy, although it is not suitable for systemic therapy of GB. It is possible that the peptide acts differently in other types of cancers, which remains to be elucidated in further studies (60).

## 3 Discussion: limitations and challenges

Bioactive peptides have ushered in a new era of targeted cancer therapies serving as a model for new drug development. The use of venom peptides offers many advantages because they have high specificity and selectivity for specific ion channels and other receptors on the plasma membrane and membranes of organelles in cells. Besides, venom peptides are also developed as tags to target cytotoxic drugs or nanoparticle scaffolds to specific cell types (6).

Venom peptides are very stable over a wide pH-range and are usually highly resistant to degradation by proteases, for example due to unusual post-translational modifications or a high number of disulfide bonds (6, 136), properties that are welcome for pharmaceutical applications. Moreover, these molecules are relatively small what facilitates their penetration into tissues and binding to molecular targets and receptors to achieve the desired therapeutic effects. Bioactive peptides have evolutionarily conserved domains that simplify further molecular modelling of cognate peptides with increased selectivity for their cellular and molecular targets in cancer in the process of selective drug development (137).

On the other hand, there are numerous serious obstacles that restrict more successful application of bioactive venom peptides in medicine. One is their pharmacology. To confine their action just on malignant cells, they have to be delivered precisely to the tumor (101), in our case to GB, more desirable specifically to GSCs. The other problem is their complexity and size. Besides being regularly post-translationally modified, bioactive peptides usually contain multiple disulphide bonds, which make their synthesis, needed when repurposed as anti-cancer drugs, complicated and expensive (136). Also a drawback, mechanisms of action of many bioactive peptides is still not clearly understood at the molecular level (20).

The success rate of the bioactive peptides to pass clinical trials is still very low. For cancer treatment only 3.4% of considered venom peptides have entered clinical trials passing phases I–III (138). However, novel methodologies are developed that give hope to improve translation from bench to bedside. Combined approaches of adding bioactive peptides to standard cancer treatments are to be explored using advanced GB *in vitro* models (139). Recent advances in the establishment of GB organoids (140, 141) that accurately recapitulate the genetic and molecular features of original tumors, including inter-patient and intra-tumor heterogeneity, are promising a brighter future.

## Author contributions

Conceptualization: BM, MN, and IK; writing—original draft preparation: BM, TL, and IK; writing—review and editing: BM, MN, TL, and IK; visualization: BM. All authors contributed to the article and approved the submitted version.

## Funding

This work was supported by the Slovenian Research Agency Programme grants, P1-0245 (to TL) and P1-0207 (to IK), and the Young Researcher grant 10040137 (to BM).

## Acknowledgments

We would like to thank Prof. Cornelis Van Noorden for critically reading the manuscript.

## Conflict of interest

The authors declare that the research was conducted in the absence of any commercial or financial relationships that could be construed as a potential conflict of interest.

## Publisher’s note

All claims expressed in this article are solely those of the authors and do not necessarily represent those of their affiliated organizations, or those of the publisher, the editors and the reviewers. Any product that may be evaluated in this article, or claim that may be made by its manufacturer, is not guaranteed or endorsed by the publisher.
